# Recruiting Hard-to-Reach Subjects for Exercise Interventions: A Multi-Centre and Multi-Stage Approach Targeting General Practitioners and Their Community-Dwelling and Mobility-Limited Patients

**DOI:** 10.3390/ijerph10126611

**Published:** 2013-11-29

**Authors:** Michael Brach, Anna Moschny, Bettina Bücker, Renate Klaaßen-Mielke, Matthias Trampisch, Stefan Wilm, Petra Platen, Timo Hinrichs

**Affiliations:** 1 Institute of Sport and Exercise Science, University of Muenster, 48149 Muenster, Germany; 2 Department of Sports Medicine and Sports Nutrition, Ruhr-University Bochum, 44801 Bochum, Germany; E-Mails: anna.moschny@rub.de (A.M.); petra.platen@rub.de (P.P.); 3 Institute of General Practice and Family Medicine, Witten/Herdecke University, 58448 Witten, Germany; E-Mail: bettina.buecker@uni-wh.de; 4 Institute of General Practice, Heinrich Heine University Duesseldorf, 40225 Duesseldorf, Germany; E-Mail: stefan.wilm@med.uni-duesseldorf.de; 5 Department of Medical Informatics, Biometry and Epidemiolgy, Ruhr-University Bochum, 44801 Bochum, Germany; E-Mails: klaassen-mielke@amib.rub.de (R.K.-M.); matthias-trampisch@amib.rub.de (M.T.); 6 Impairment Control, Capacity Building & Health Maintenance Unit, Swiss Paraplegic Research, 6207 Nottwil, Switzerland; E-Mail: timo.hinrichs@paraplegie.ch

**Keywords:** general practitioner, recruitment, mobility limitation, exercise, older adults, selection bias

## Abstract

The general practitioner (GP)’s practice appears to be an ideal venue for recruiting community-dwelling older adults with limited mobility. This study (Current Controlled Trials ISRCTN17727272) aimed at evaluating the recruiting process used for a multi-centre exercise intervention (HOMEfit). Each of six steps resulted in an absolute number of patients (N1–N6). Sex and age (for N4–N6) and reasons for dropping out were assessed. Patient database screening (N1–N3) at 15 GP practices yielded N1 = 5,990 patients aged 70 and above who had visited their GP within the past 6 months, N2 = 5,467 after exclusion of institutionalised patients, N3 = 1,545 patients eligible. Using a pre-defined limitation algorithm in order to conserve the practices’ resources resulted in N4 = 1,214 patients (80.3 ± 5.6 years, 68% female), who were then officially invited to the final assessment of eligibility at the GP’s practice. N5 = 434 patients (79.5 ± 5.4 years, 69% female) attended the practice screening (n = 13 of whom had not received an official invitation). Finally, N6 = 209 (79.8 ± 5.2 years, 74% female) were randomised after they were judged eligible and had given their written informed consent to participate in the randomised controlled trial (overall recruitment rate: 4.4%). The general strategy of utilising a GP’s practice to recruit the target group proved beneficial. The data and experiences presented here can help planners of future exercise-intervention studies.

## 1. Introduction

Recruiting older adults to participate in exercise intervention trials has been described as challenging. For example, less than one-third of 114 trials reviewed by Campbell *et al.* [1] reached their target number of participants within the time originally specified. Recruiting problems are especially true for home-dwelling seniors who are chronically ill and have limited mobility. They would derive great benefits from preventive group exercise [2,3,4,5,6,7,8], especially when certain quality criteria are met [7,8]. However, it is often difficult for them to attend classes, which diminishes their motivation [9,10,11]. Members of the target group are generally more likely to be sedentary [12]. In consequence, it is difficult to reach them for interventional studies. 

On the other hand, even mobility-limited seniors usually visit their general practitioners (GP). We utilise this fact as a chance to approach the target group through GP practices, invite them to perform home-based exercise and offer them support. This approach has been discussed and studied recently [13,14].

While this approach is promising, it gives rise to problems of its own. Considerable efforts are required to contact and persuade GPs to participate in the study and to ensure their adherence to the methodological procedures [15]. In addition, the return in terms of eligible patients seems to be rather low. Furthermore, it has been criticised that authors of scientific articles frequently fail to report recruiting accurately [16], especially when problems occur.

The example presented by Sanders *et al.* [17] is valuable due to its uncharacteristic frankness and details. The authors documented the recruitment of over 2,000 women aged 70 and older for a study targeting fall risk. During most of the recruitment process, several recruitment methods were used with fairly little success (the method involving GPs proved to be least successful). In the end, targeted mail-outs proved successful, allowing the researchers to recruit the desired number of participants shortly before the recruiting period was scheduled to end. These results are interesting, since we combined both their least and their most effective recruitment strategies, *i.e.*, (a) involving GP practices to form a base for patient recruitment as well as for supporting the exercise programme, and (b) utilising targeted mail-outs after pre-screening patient databases.

The success of both strategies is supported by other studies as well: in a study comparing different recruitment strategies [18], recruitment utilising a GP database was most successful. Although the target group was different (women with stress/depression), the results appear to be transferable. This also is in line with a consensus report on preventive interventional trials in older persons [19]. Further recommendations include (a) screening for eligibility using a multi-stage process, (b) orientating exclusion criteria towards factors that prevent participation and avoiding exclusion due to comorbidity, (c) presenting details on attrition at each stage, including eligibility screening, enrolment and consenting. We followed these recommendations in our study (see below). The aims of the present study are (a) to report and to evaluate the recruiting process used for an intervention study using this approach (HOMEfit), and (b) to provide supportive information for planning and monitoring recruitment in similar studies.

## 2. Subjects and Methods

### 2.1. Ethics Statements and Registration

The HOMEfit study protocol was approved by the Witten/Herdecke University Ethics Committee on 15 August 2011 (Reg.-No. 77/2011) and was published recently [14]. The study was in compliance with the Helsinki Declaration. It has been registered at Current Controlled Trials (ISRCTN17727272).

### 2.2. Research Plan and Parent Study

The present study is part of a multi-step research and development plan. The object of this plan is a home-based exercise programme targeting mobility-limited and chronically ill older adults who live in their own homes. The main approach of this programme called HOMEfit is to contact and to support participants through their GP’s practice. To this end, an exercise therapist cooperates with local GP practices for patient motivation, information, exercise quality and behavioural change.

The claim of our research and development plan is to perform research as rigorously as in medical drug development. As a consequence of this, we follow the framework proposed by the UK Medical Research Council (MRC) for evaluating complex interventions [20]. The MRC framework comprises four phases: We started with a phase of development with literature and survey research [21,22,23]. During the feasibility phase, all previously set quantitative feasibility criteria were met in a formal feasibility study [24], although further improvement was required for recruitment documentation and quality (see below).

For this reason, we decided to start the evaluation phase [25] by conducting a two-arm interventional randomised controlled trial (RCT). Recruitment for this RCT is subject of the present article. A future fourth phase would comprise comprehensive implementation of the HOMEfit programme.

The primary outcome of the RCT was functional strength measured by the chair-rise test [26]. The corresponding sample size and power calculations yielded 210 as appropriate target number of participants [14]. Inclusion criteria of the RCT were age 70 years and above, home-dwelling, at least one chronic disease as defined by the International Classification of Diseases (ICD) and problems with mobility as defined by the International Classification of Functioning, Disability and Health (ICF). Exclusion criteria comprised several practical, medical and safety aspects. Exclusion of subjects who were not able to perform the chair-rise test had to be ascertained, because otherwise assessment of the primary outcome would be impossible. In order to reach the target group, patients reporting: (a) regular exercises, sporting activities or leisure activities that cause sweating and/or harder breathing for 2 hours or more per week or (b) outdoor walks for 4 hours or more per week had to be excluded.

The revised version of the 12-week-programme contained: (a) behavioural strategies, (b) exercises targeting strength, balance and flexibility and (c) brisk walking. The control group received instructions for baseline physical activities, defined as “light-intensity activities of daily life, such as standing, walking slowly, and lifting lightweight objects” [27] (p. 2), without increasing intensity.

Participants in both groups had two practice assessments and two telephone interviews with the blinded outcome assessor, as well as three telephone consultations and five personal appointments with the exercise therapist. 

### 2.3. Recruiting GP Practices, Recruiting Therapists

The GP practices belong to a network of “research practices” administered by the Institute of General Practice and Family Medicine, Witten/Herdecke University. They were recruited with an official invitation letter from the study physician, using several criteria, the main one being the establishment of an electronically searchable patient database. Exercise therapists involved in the study had completed formal vocational training or academic higher education of at least three years. These and other recruitment details can be found in the study protocol [14].

In the feasibility study, it was nearly impossible to analyse recruitment quantitatively, because data on patients screened for eligibility and/or invited to the detailed information session with the exercise therapist were often incomplete or were of low quality (e.g., GPs had filled in data sheets from memory or not until several days after seeing the patient). The research team conjectured that GPs may not have been aware of the importance of keeping records or that the GPs were unable to rigorously record information beyond that associated with the patients’ actual participation. By mentioning these problems, we do not intend to blame the GPs, since these findings are consistent with a comprehensive analysis performed by the Clinical Trials in German General Practice Network. [28]. Therefore, for the RCT, we considered changing the methodology. In addition to the letter of invitation, in order to foster a collegial atmosphere, the study physician personally visited each centre to brief the GP before the exercise therapist implemented procedures.

### 2.4. Recruiting Participants

The feasibility study protocol [24] had assigned the GP to identify (regarding age and medical eligibility criteria), inform and invite potential participants during normal consultation hours in the order in which the patients were regularly scheduled for their appointments. The GP was requested to send each subject to an exercise therapist who was present in a separate room for the entire recruiting period. The therapist was in charge of assessing further eligibility, informing eligible participants and getting written informed consent. The recruiting period for a given centre ended when the planned number of patients was formally included in the study.

Extending the recruiting period until the planned number was reached was clearly too inefficient for recruiting the number of patients needed for an RCT. Another aspect had to be improved as well: in the feasibility study, the initial identification, screening and invitation of potential participants took place during regular patient consultations, which made it difficult to describe in a standardised manner and also posed an unnecessary burden on the GP.

**Figure 1:**
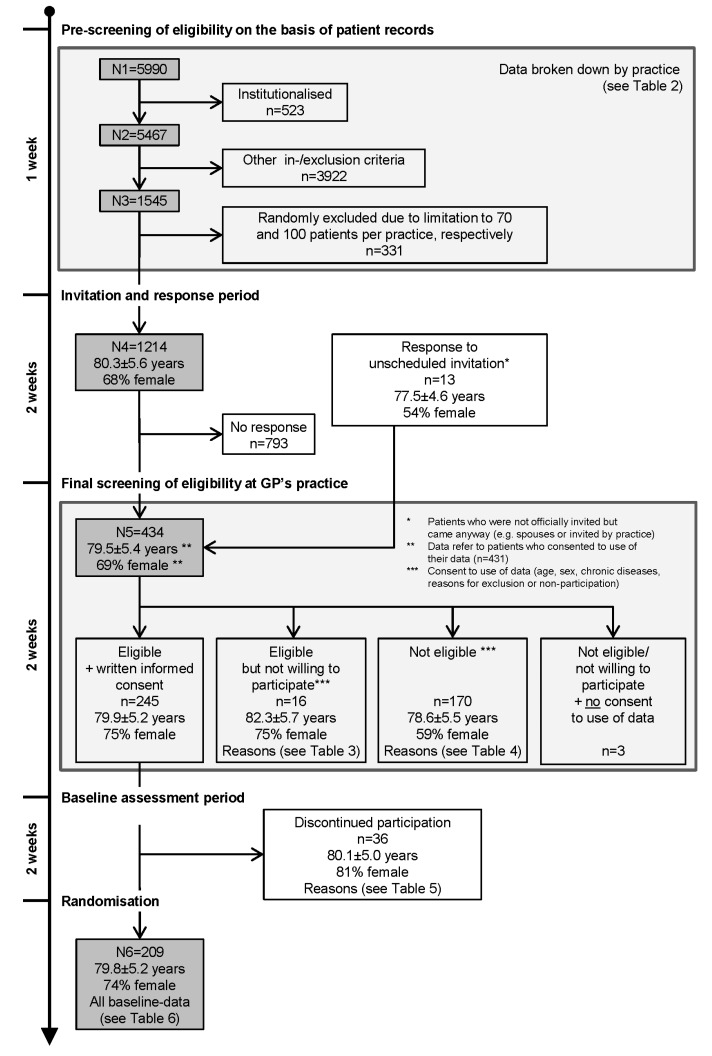
Recruiting process.

Consequently, recruiting was divided into several distinct steps, starting with pre-screening of electronic patient databases and records, performed by practice nurses together with the study physician. They asked the GPs only to clarify special cases. Parameters N1 to N6 were defined for the purpose of ongoing control of recruitment success as well as for posthoc evaluation. Table 1 and Figure 1 present overviews of parameters and time flow, respectively.

**Table 1:** Recruitment steps and parameters.

Step	Parameter and/or activity
Searching the patient database	N1: no. of patients ≥ 70 years who have seen their GP within the past 6 months
Screening clinical records I	N2: no. of community-dwelling patients out of N1
Screening clinical records II	N3: no. of eligible patients out of N2 (inclusion and exclusion criteria). If N3 < 20, the respective centre is excluded
Compiling the final invitation list	N4: no. of entries (potentially random selection out of N3, depending on response rate)
Invitation	Mailing letters, making appointments
Final eligibility, study information, consent	N5: no. of patients attending the practice appointment
Baseline data	N6: no. of patients out of N5 who keep their first appointment with the exercise therapist
Intervention start	

### 2.5. Limitation Algorithm Set at Centre Level

Due to limited resources of the participating practitioners, the maximum number of patients included in the study was limited to 20 per practice. Care was taken to avoid turning down patients who had responded to an invitation and were eligible. Therefore, a limitation algorithm regarding the number of invitations to be mailed was established in order to go below the limit of 20 actual participants: x potential participants were randomly selected from N3 (the number of eligible patients after screening of the medical records) and invited (*i.e.*, N4 set to x), if N3 > x. If N3 < 20, the centre was excluded from the study (*i.e.*, N4 set to zero) in order to conserve resources of the research team. Otherwise, all eligible patients were invited (*i.e.*, N4 set to N3). Response rates and outcomes of the final eligibility screening were carefully observed and recruitment success was extrapolated in order to adapt x within the course of the study if necessary to reach the target number.

## 3. Results

A letter of invitation was sent to 53 general practices, followed by telephone calls by the study physician. 10 GPs were continuously not available, 43 received calls and personal visits by the study physician. 28 out of those 43 GPs refused participation or were not eligible (main reasons: no interest, no time, separate room for exercise therapist visits not available or not adequate). In total, 15 GP practices were included as study centres.

Participating practices were located in urban (n = 11; 73%) and rural (n = 4; 27%) areas. They were categorised as small (n = 2; 13%; fewer than 900 patients treated quarterly), medium (n = 8; 53%; 900–1,500 patients) or large (n = 5; 33%; more than 1,500 patients) and were run by one (n = 7; 47%), two (n = 6; 40%) or three (n = 2; 13%) practitioners. One (n = 7; 47%) or two (n = 8; 53%) practitioners per practice took part in the study. Ten practitioners were female (43%) and 13 were male (57%). The age range was 38 to 59 years (49.5 ± 6.3 years).

Figure 1 shows a flow chart of the recruiting process discussed in the present paper. It may serve as a roadmap for the tables presented in this section. The recruitment of participants started in December 2011 (first screening of patient records) and ended in December 2012 (last patient randomised). The recruitment cycles lasted six to eight weeks per centre, with an overlap of one to seven weeks.

### 3.1. From Pre-Screening of Eligibility to Invitation and Response

The patient databases at the fifteen GP practices contained N1 = 5,990 patients aged 70 years and above. After excluding 523 institutionalised patients, the records of N2 = 5,467 patients were screened for other inclusion and exclusion criteria, yielding N3 = 1,545.

The limitation algorithm described in the methods section started with an upper limit of x = 70 invitations in order to target a mean of 14 inclusions per practice. The recruitment success (number of final study participants out of the number of invitation letters) was estimated by response rates and assessment success rates for each of the centres (see Table 2). This was extrapolated to all centres.

**Table 2:** Recruitment parameters N1–N6 and success rates broken down by practice.

Practice (P)	N1	N2	N3	N4	N5	N6	N3/N1 * (%)	N5/N4 ** (%)	N6/N5 *** (%)
1	378	333	278	70 ^§^	24	11	73.5	34.3	45.8
2	537	460	70	70	23	11	13.0	32.9	47.8
3	384	352	57	57	25	16	14.8	43.9	64.0
4	546	532	143	70 ^§^	38	17	26.2	54.3	44.7
5	611	529	120	70 ^§^	24	9	19.6	34.3	37.5
6	359	338	100	100	34	16	27.9	34.0	47.1
7 ^†^	720	684	86	86	31	16	11.9	36.0	51.6
8 ^†^	348	319	90	90	33	10	25.9	36.7	30.3
9	279	248	65	65	23	13	23.3	35.4	56.5
10 ^†^	230	226	74	74	19	13	32.2	25.7	68.4
11	392	326	97	97	32	16	24.7	33.0	50.0
12	416	401	87	87	28	15	20.9	32.2	53.6
13 ^†^	483	433	93	93	36	18	19.3	38.7	50.0
14	192	172	85	85	33	17	44.3	38.8	51.5
15	115	114	100	100	31	11	87.0	31.0	35.5
**Total**	**5,990**	**5,467**	**1,545**	**1,214**	**434**	**209**	**25.8**	**35.7**	**48.2**

*Notes:* The upper limit for the number of invitations was set to n = 70 for P1–P5. After interim analysis of recruitment success, the upper limit was increased to n = 100 for P6–P15. ^§^ Randomly selected from N3 to be invited; ^†^ GP practice located in rural (as opposed to urban) area; * pre-screening success rate; ** response rate (includes unscheduled response from 13 patients, see Figure 1); *** final eligibility screening success rate (including non-participants not allowing data usage).

An interim analysis of the completed recruitment steps in the first five practices indicated that the total of 210 participants targeted was unlikely to be reached. Therefore, the upper invitation limit was set to 100 invitations per practice for the ten remaining practices. In total, 331 records from three practices were randomly excluded. For each centre yielding more than the lower limit (N3 ≥ 20), no centre had to be excluded.

The pre-screening and selection process resulted in N4 = 1,214 patients aged 80.3 ± 5.6 years (mean ± standard deviation); 68% were female. They were officially invited to the GP’s practice for final assessment of eligibility.

In Table 2, the screening and selection numbers are broken down by centre. Practice numbers (column 1) are listed in order of recruitment start. Columns two to seven show recruitment numbers as described above. The pre-screening success rate of 25.8% (column eight, N3/N1, range 11.9%–87.0%) describes the yield of the pre-screening phase. Using the response rate of 35.7% (column nine, N5/N4, range 25.7%–54.3%) and the final eligibility screening success rate of 48.2% (column ten, N6/N5, range 30.3%–68.4%), the overall recruitment success per centre can be evaluated by multiplying the three above-mentioned parameters. The overall rates of patients included per centre in relation to all patients in the age group of this centre vary from 2.0% to 11.6% (overall recruitment rate: 4.4%).

### 3.2. Final Screening of Eligibility at GP’s Practice

Four hundred and thirty four (N5) patients were personally screened by the exercise therapist with regard to inclusion and exclusion criteria. The number includes 13 persons who responded to an unscheduled invitation by the practice, or who were spouses who appeared instead of the patient invited.

Each person was informed about the study, and if eligible, was asked for written consent to participate. Subjects who were ineligible or not willing to participate were asked to allow the researchers to use assessment data for statistics. Since three patients refused usage of their data, the following figures refer to the N5 − 3 = 431 patients who consented to use of their data.

Two hundred and sixty one subjects (60.6%) were eligible. Sixteen (6.1%) of the eligible subjects refused to participate and were asked to state their reasons for refusal (see Table 3). The most frequently mentioned reason (by six subjects) was time constraints. Two hundred and forty five subjects gave written informed consent and were invited for baseline assessments (see below).

**Table 3:** Eligible patients not willing to participate: reasons for non-participation (multiple answers allowed).

Reasons	Total (n = 16)		Men (n = 4)		Women (n = 12)	
	n	%	n	%	n	%
Health concerns	3	18.8	2	50.0	1	8.3
No interest	3	18.8	1	25.0	2	16.7
No time	6	37.5	1	25.0	5	41.7
Expected effort too high	3	18.8	2	50.0	1	8.3
Rejection of telephone calls and/or assessments	3	18.8	1	25.0	2	16.7
Other reasons *	4	25.0	0	0.0	4	33.3

* Other reasons were (each n = 1): Still feeling too active, expectations regarding study not met, time commitment not wanted, wish for more information regarding the two interventions.

The exclusion reasons applying to 170 subjects are shown in Table 4. The main reasons were physical activity levels that were too high and lack of mobility limitation, which applied to 52% and 34% of the subjects excluded. Both reasons refer to the target group of the exercise programme. 31.2% of the excluded subjects (12.2% of patients assessed) could not perform the chair-rise test and had to be excluded for a methodological reason although they belonged to the target group of the exercise programme.

**Table 4:** Reasons for non-eligibility as determined during the screening at the GP’s practice (multiple answers allowed).

Reasons	Total (n = 170)		Men (n = 69)		Women (n = 101)	
	n	%	n	%	n	%
No medical clearance	13	7.6	5	7.2	8	7.9
Not affected by defined chronic diseases	0	0.0	0	0.0	0	0.0
No mobility limitation	58	34.1	25	36.2	33	32.7
Physical activity level too high	88	51.8	39	56.5	49	48.5
Inability to perform the chair-rise test	53	31.2	18	26.1	35	34.7
Inability to participate in the proposed course of intervention	20	11.8	8	11.6	12	11.9
Other criteria	22	12.9	16	23.2	6	5.9

**Table 5:** Reasons for discontinued participation before randomisation.

Reasons		Total (n = 36)		Men (n = 7)			Women (n = 29)	
		n	%		n	%	n	%
**Exclusion by GP, therapist or SAE-manager due to (S)AE**		**1**	**2.8**		**0**	**0.0**	**1**	**3.4**
**Exclusion by GP or therapist due to a posteriori detection of other exclusion criteria **		**9**	**25.0**		**2**	**28.6**	**7**	**24.1**
	Inability to participate in the proposed course of intervention	2			0		2	
	Chair-rise test not feasible during baseline assessment	3			2		1	
	Planned hospitalisation	2			0		2	
	Other *	2			0		2	
**Patient’s decision to discontinue (multiple answers allowed)**		**26**	**72.2**		**5**	**71.4**	**21**	**72.4**
	Health concerns due to (S)AE (without exclusion by GP, therapist or SAE-manager)	4			0		4	
	Health concerns without (S)AE	4			3		1	
	No longer interested	4			1		3	
	No more time	9			2		7	
	Expected effort too high	2			0		2	
	Death in the family	2			0		2	
	Other **	8			1		7	

(S)AE: (Severe) adverse event; GP: general practitioner. * Other exclusion reasons were: no telephone calls possible, reason not stated; ** Other reasons were: brief hospitalisation; telephone consultations not wanted; questions during baseline telephone interview too personal; personal reasons; foot problems, planned inpatient assessment, programme too demanding.

Overall, more women than men were eligible. Reviewing the most frequent reasons for exclusion, men had a higher percentage of exclusion for being too active (M *vs.* F, n = 39 *vs.* 49, 29% *vs.* 17% of subjects assessed) and/or not having a mobility limitation (n = 25 *vs.* 33, 19% *vs.* 11%). There was no relevant difference between men and women with regard to exclusion due to inability to perform the chair-rise test (n = 18 *vs.* 35, 13% *vs.* 12%).

Thirty-six eligible patients dropped out during the two-week period between consent to participate and randomisation. The reasons are presented in Table 5. Ten subjects had to be excluded by the GP, the therapist or the SAE-manager due to a (severe) adverse event (S)AE (n = 1) or due to a posteriori detection of other exclusion criteria (n = 9). 26 subjects withdrew of their own accord. For these cases, time constraints were again the most frequently stated reason.

### 3.3. Baseline Characteristics of Randomised Study Participants

Two hundred and nine (N6) subjects (79.8 ± 5.2 years, median 80 years, 74% female) were randomised after being judged eligible, giving their written informed consent to participate in the RCT, and undergoing baseline measurements. They reached the final stage of the recruitment procedure and will be called participants of the study.

Baseline measurements are presented in Table 6. The age of the participants ranged from 70 to 94 (median 80, mean ± SD 79.8 ± 5.2, n = 154) years in women and from 71 to 90 (median 81, mean ± SD 79.8 ± 5.3, n = 55) years in men, respectively. Since the presence of defined diseases was recorded during the final eligibility screening, a comparison of study participants with disjoint partial samples in the recruitment course (not eligible, eligible but not willing, withdrawal before randomisation) is possible. The percentage of subjects in these partial samples with four or more of the diseases varied from 38% to 45% (participants: 36%). In all groups, the main diagnoses were essential hypertension (87% to 94% in all groups, participants: 90%), spinal osteochondrosis (61% to 69%, participants: 68%), and gonarthrosis (49% to 69%, participants: 60%).

**Table 6:** Patient characteristics at baseline.

Characteristics				Total (n = 209)		Men (n = 55)		Women (n = 154)	
				n ^†^		n ^†^		n ^†^	
**Socio-demographic data (%)**									
	Socio-economic status			172		49		123	
		low			38.4		18.4		46.3
		middle			52.3		65.3		47.2
		high			9.3		16.3		6.5
	Household size			207		54		153	
		1			58.9		22.2		71.9
		2			37.2		70.4		25.5
		3 or more			3.9		7.4		2.6
	Contact to relatives, friends, acquaintances (per week)			205		53		152	
		0 times			30.7		32.1		30.3
		1–3 times			62.0		66.0		60.5
		≥4 times			7.3		1.9		9.2
**Anthropometric data (mean ± SD)**									
	Height (cm)			209	163.6 ± 9.4	55	174.7 ± 6.2	154	159.7 ± 6.8
	Weight (kg)			208	82.4 ± 19.0	55	94.0 ± 23.0	153	78.2 ± 15.4
	BMI (kg/cm²)			208	30.6 ± 5.7	55	30.7 ± 6.4	153	30.6 ± 5.5
	Waist circumference (cm)			209	105.7 ± 14.5	55	112.4 ± 16.6	154	103.3 ± 12.9
**Physical activity ^#^ (median time/week)**									
	Housework (hh:mm)			195	9:00	55	3:30	140	12:15
	Sporting activity (hh:mm)			206	0:13	54	0:30	152	0:10
	Walking for leisure (hh:mm)			202	3:00	53	3:30	149	3:00
	Gardening (hh:mm)			199	0:15	53	0:00	146	0:15
	Total ^β^ (hh:mm)			185	17:00	50	13:03	135	18:15
**Chronic diseases (%)**				209		55		154	
	Essential hypertension				90.4		90.9		90.3
	Chronic ischaemic heart disease				29.2		50.9		21.4
	Chronic heart failure				33.5		54.5		26.0
	Type 2 diabetes				39.7		45.5		37.7
	Peripheral arterial disease				12.0		29.1		5.8
	COPD				22.5		34.5		18.2
	Chronic kidney disease				17.7		29.1		13.6
	Spinal osteochondrosis				68.4		60.0		71.4
	Coxarthrosis				46.4		43.6		47.4
	Gonarthrosis				60.3		58.2		61.0
	Osteoporosis ^§^				21.1		5.5		26.6
	Number of specified chronic diseases								
		1			4.3		5.5		3.9
		2–3			32.1		21.8		35.7
		4–5			35.9		25.5		39.6
		≥6			27.8		47.3		20.8
**Other health-related factors (%)**									
	Need for walking aid			206	54.4	55	52.7	151	55.0
	Falls (past 12 months)			204		54		150	
		0			72.1		74.1		71.3
		1			15.7		14.8		16.0
		2			5.4		5.6		5.3
		≥3			6.9		5.6		7.3

^†^ Number of cases with complete data per item; ^#^ assessed with PRISCUS-Physical Activity Questionnaire (PAQ); ^β^ sum of above-mentioned activity categories; ^§^ with or without pathologic fracture; COPD = chronic obstructive pulmonary disease; SD = standard deviation.

## 4. Discussion

In order to derive supportive information for planning and monitoring recruitment of future studies utilising similar approaches, we evaluated the recruiting process of an exercise intervention study and described the multi-stage and multi-centre procedures and their outcomes in detail. The target group—community-dwelling, chronically ill and mobility-limited older adults—was approached by screening 5,990 patient database entries at 15 GP practices and inviting 1,214 patients for personal eligibility assessment. In the end, 209 subjects were randomised.

### 4.1. Review of Recruitment Methods, Outcome and Attrition

#### 4.1.1. Recruitment Outcome

The target number of 210 participants was nearly reached (N6 = 209). Only 6% (n = 16) of the eligible subjects were not willing to participate. However, another 10% (n = 26) withdrew their participation during the two weeks between baseline assessment and randomisation, resulting in a randomisation rate of 17.2% (overall response rate 35.7%, multiplied by the overall randomisation rate 48.2%, see Table 2).

After pre-screening electronic databases, in a similar study, Campbell *et al.* [29] asked GPs from 17 practices to invite patients to a strength and balance home exercise programme and reached a much higher randomisation rate of 37.5%. Contrary to the present study, invited patients were visited by the practice nurse and there were fewer exclusion criteria. The different steps of recruitment (database, invitation, procedure used during the visit) are not fully described in detail in the Campbell paper, which limits comparability.

Research teams led by Stevens [30] and Munro [31] assessed eligibility using an initial questionnaire, then randomised all eligible patients for an exercise intervention (Stevens *et al.*: 10 weeks combining leisure centre and home-based activities, Munro *et al.*: free exercise classes over a period of two years). While they received different responses (35% *vs.* 82%) and different eligibility rates (32% *vs.* 80%), overall recruitment rates were similar (11% *vs.* 17%, calculated from the figures presented).

These examples show that recruitment outcomes are difficult to compare, even when similar approaches are used. In order to make trials comparable, interim outcomes of single recruitment steps should be reported [19]. Higher rates are not always “better”. For example, eligibility rates heavily depend on inclusion and exclusion criteria. Eligibility outcomes of database searches used for pre-screening, should not be judged by their sheer quantity, but by their influence on receiving high final eligibility rates during personal assessment. This reduces unnecessary effort on the part of ineligible subjects and research staff. If different recruitment strategies are used, potential selection bias should be checked and corrected as necessary [32]. In the present study, exclusion during personal assessment mainly occurred for mobility- and activity-related reasons, *i.e.*, methodological reasons, since medical aspects had been assessed from patient records previously.

#### 4.1.2. The Final Sample

Inclusion and exclusion criteria serve as operationalisation of a certain target group. Therefore, it is useful to compare the final sample to sample characteristics of studies targeting on similar populations. The following paragraphs discuss, in how far the target group has been reached beyond inclusion and exclusion criteria, namely in terms of typical diseases and limited mobility. 

The socio-economic status of the female sample was shifted slightly from medium to lower status, compared to representative data regarding the 75–79 year-old population [33,34]. Since the mean age in our sample is 80 years, this is in line with the tendency of older female populations to have a lower economic status. The male sample is well within the 95% confidence of the population sample.

Several features may be compared to a larger sample of primary care patients studied by Moschny *et al.* [23,10]. While the age is similar (median 77, range 72–93 years, n = 1,610) to our sample (median 80, range 70–94), the percentage of participants living alone (16%/54% men/women) is higher in the present sample (22%/72%). This also applies to the percentage of participants in need of a walking aid (14%/18% *vs.* 53%/55%), participants with a fall history in the last 12 months (17%/25% *vs.* 26/29%), as well as rates of each disease (Table 6). The main fall risks, such as osteoarthritis (relative frequency in our sample >60%, RR/OR for falls 2.4, according to international fall prevention guidelines [35]), need for walking aids (54%, RR/OR 2.6), one or more falls during the past 12 months (28%, RR/OR 3.0), were also distributed throughout the sample.

In summary, we succeeded in recruiting a sample with typical features of our target group, and with some focus on mobility and corresponding impairments, making them hard to reach for study participation and for exercise intervention. Thus, the sample comprises “subjects at high risk of developing disability and likely to benefit from the intervention”, as has been recommended for inclusion in interventional trials [19].

There is a definite limitation regarding the target group of our study. 12.2% of the subjects could not perform the chair-rise test. Since this test had been selected as the primary outcome, there was no alternative to establishing a corresponding exclusion criterion, although these subjects could also have benefited from the exercise programme. Future research on test methods, e.g., using technical test equipment, may reduce exclusion due to choice of outcome measures [36,37]. On the other hand, subjects who have already developed the disability that the preventive exercise was intended to prevent may reduce the statistical test power [19].

#### 4.1.3. Recruiting Procedures

In the Methods section of this article, some changes to the feasibility study were justified and applied. These relate to the initial eligibility screening using electronic databases (Section 2.3), and the stepwise organisation of the recruitment process including written invitation (Section 2.4) As a consequence, two main advantages were experienced in the present study.

First, during the feasibility study, the GP had been solely in charge of both patient invitation and eligibility, patient selection and data quality—in addition to the treatment of the patient. In course of the present study, this was changed to an initial database search for eligible patients (performed by a practice nurse and the study physician), which also utilised the GP’s medical competence, but in an objective manner, and without time pressure of daily office hours. In addition, the documentation of the recruitment process was ensured.

Second, the exercise therapist was no longer dependent on the GP sending the next interested and eligible patient as he/she had been in the feasibility study. Since appointments were organised by the practice nurses, the exercise therapist could work during fixed office hours and had very little unproductive waiting time, and this was also true for the patients. Practice nurses were more extensively engaged than during the feasibility study, although they were engaged in typical activities such as organising and communicating with patients and other health care professionals.

In summary, the professionals involved (GPs, exercise therapists, practice nurses), concentrated on their own competence and/or their resources were conserved. Recruitment documentation and data quality were significantly improved.

A limitation of our study is the fact that recruitment and implementation at the centre level was not the subject of research. In fact, we worked with a network of general practitioners who were generally interested in taking part in research. As soon as evidence is in place for the programme’s effectiveness at the patient level, future studies will have to prove our approach with regard to centre recruitment. According to Williamson *et al.* [15] “enablers” to recruitment may include the quality of an existing database, a letter of invitation, an appealing topic, minimal time commitment, or professional training credits.

#### 4.1.4. Attrition and Bias during the Recruitment Steps

The multi-step recruitment yields an opportunity to look for possible bias caused by potential participants dropping out or being excluded. This relates to information on age, sex and health status, which was recorded during early steps. The number and type of diseases of the subjects seem to be stable throughout the recruitment process. Some information is available on subjects who did not take part in the study: some were eligible but refused to participate (Figure 1), while others discontinued participation between baseline measurements and the time of randomisation. Harris [38] found that participation (*vs.* non-participation) corresponded with lower health status, male sex, but not with age. Our data support the correspondence with age as well as the increase in the rate of male participants over the last recruitment steps (consenting, baseline assessments, randomisation). The health status of eligible subjects who refused to participate was relatively low: 44% had six or more defined chronic diseases, compared to 27% of willing subjects. In conclusion, the time point of refusal or withdrawal could contribute to possible bias. This corresponds to the above-mentioned study by Moschny *et al.* [10], in which the target group most frequently considered poor health a barrier to physical activity, as did participants aged 80+ years in particular. An approach of Harris *et al.* [38,39] could help to better estimate these loss risks and has potential to recoup its costs. They asked non-participants to fill a questionnaire about their health, physical activity levels and reasons for not wanting to participate.

With regard to the per-centre schedule of the study, the recruitment phase takes considerable time compared to the 12-week intervention length. Thus, apart from certain reasons or barriers, the sheer duration may also be a factor for attrition. This will be tested by means of a drop-out analysis to be performed in the course of intervention outcome analyses.

### 4.2. Supportive Information for Recruiting GP Patients

The findings of the present study may be useful: (a) for planning future studies using similar approaches and (b) for monitoring recruitment for an ongoing study.

The pre-screening success rates reported in the results section may help to roughly plan the number of centres needed and the number of invitations to send to pre-screened patients. In fact, our data correspond to a recently published study protocol for a walking intervention [39]. The authors plan to conduct the same recruitment steps as we did in our study, with the exception that they cooperate with three much larger (list size of at least 10,000 patients) GP practices. For this reason there is no limitation algorithm but a plan for sending several rounds of invitation letters. Our data (36% response, 17% recruitment success) support the expected rate of 10%–40% given in the study protocol. However, Harris *et al.* do not consider a loss between baseline assessment and start of the intervention, although they include an additional seven-day physical activity measurement.

In order to evaluate the limitation algorithm applied before compiling the final invitation list of the present study, we refer to ranges (see Table 2) instead of means and standard deviations, because adaptation decisions from centre to centre have to be taken based on limited figures, without knowledge of the sample. The pre-screening success rates mentioned above show a wide range (12% to 87%) and, consequently, this is also true for the overall recruitment success rate (2% to 12%). Therefore, neither parameter is useful for controlling and adapting ongoing recruitment. In contrast, the response rate (26% to 54%) and eligibility success rate (30% to 68%) were far more stable, and are therefore useful for control purposes, as has been shown in the present study. In summary, the limitation algorithm has proved its usefulness for securing recruitment and resource allocation by estimating the yield at centre level. Using sex-specific anticipated rates for response and consenting could improve the recruitment results.

In order to fine-tune planning and monitoring, known point-of-time-specific and sex-specific barriers as well as risks for bias mentioned above should be considered. Consequently, progress should culminate in probability modelling of recruitment results [40] and should take into account the knowledge at start as well as the information gained during implementation.

## 5. Conclusions

The use of a GP electronic database has again proven to be an effective tool for pre-screening eligibility of hard-to-reach subjects with a low rate of false positives (Table 4). Since it contains postal addresses, it is also efficient for initially approaching corresponding target groups through the GP. There are different options for further contact, such as home visits [29], telephone follow-up [18] or by eliciting patients’ responses when they request a practice appointment. Having to keep an appointment at the practice ensured soft inclusion criteria in the present study, such as the subjects’ ability to organise their lives and ability to visit the practice.

Pre-trial recruitment planning and on-trial recruitment monitoring can be fostered by: (a) using benchmarks and estimates from similar studies and (b) in the case of multi-centre trials, defining an algorithm at centre level. Adapting recruitment strategies (e.g., the upper limit for invitations) was important in reaching the desired target. Further research and development should comprise sex-specific estimators, possible barriers to participation and reasons for withdrawal at different potentially critical points in time. Independent from factual details, following a research framework containing a distinct feasibility phase was important in order to form the recruitment strategy. 
